# Relationship between hippocampal volume and treatment response before and after escitalopram administration in patients with depression

**DOI:** 10.1038/s41398-025-03796-4

**Published:** 2025-12-30

**Authors:** Toshiharu kamishikiryo, Eri itai, Yuki mitsuyama, Yoshikazu masuda, Osamu yamamoto, Tatsuji tamura, Hiroaki jitsuiki, Akio mantani, Norio yokota, Go okada

**Affiliations:** 1 Department of Psychiatry and Neurosciences, Graduate School of Biomedical and Health Sciences, Hiroshima University, Hiroshima, Japan; 2 Ujina Mental Clinic, Hiroshima, Japan; 3 Tamura Mental Clinic, Hiroshima, Japan; 4 Jitsuiki Clinic, Hiroshima, Japan; 5 Mantani Mental Clinic, Hiroshima, Japan; 6 Yokota Mental Clinic, Hiroshima, Japan

**Keywords:** Depression, Neuroscience

## Abstract

The hippocampus plays a crucial role in memory formation and emotional regulation, and is closely related to the pathology of depression. Escitalopram, a selective serotonin reuptake inhibitor, promotes neurogenesis in the dentate gyrus of the hippocampus and alters its structure. This study examined the relationship between these escitalopram-induced structural changes and treatment response in 107 patients with moderate to severe depression, of whom 56 were female (52.3%), with a mean age of 41.5 years (range: 25–73 years). Follow-up data were available for 71 patients (66.4%). Changes in the volume and laterality of hippocampal subregions were compared before and after escitalopram treatment between Responders to escitalopram (*n* = 53) and Nonresponders (*n* = 54). The results indicated that Responders had a larger left hippocampal volume (β = 189.3, 95% CI [71.4, 307.2], Cohen’s f² ≈ 0.094) and greater leftward laterality (β = 0.012, 95% CI [2.2 × 10⁻⁴, 0.024], Cohen’s f² ≈ 0.04) than Nonresponders at baseline. Additionally, the right hippocampus and right hippocampal head exhibited increased volume (β = 80.6, 95% CI [28.5, 132.8], Cohen’s f² ≈ 0.005; β = 50.4, 95% CI [22.2, 78.5], Cohen’s f² ≈ 0.006) and altered laterality (β = −0.011, 95% CI [−0.022, −4.1 × 10⁻⁴], Cohen’s f² ≈ 0.003; β = −3.3 × 10⁻⁴, 95% CI [−5.7 × 10⁻⁴, −8.3 × 10⁻⁵], Cohen’s f² ≈ 0.006) in response to escitalopram in Responders compared to Nonresponders. Furthermore, the volume changes in the right hippocampus and right hippocampal head correlated with core depressive symptoms. It is suggested that these volume changes play an important role in the improvement of core symptoms of depression by escitalopram treatment. These findings can help to reveal the mechanisms of action of selective serotonin reuptake inhibitors and have the potential to facilitate the early identification of patients who will respond to treatment.

## Introduction

Major depressive disorder (MDD) is considered a heterogeneous disorder with a variety of symptoms [1]. Antidepressant medication is recommended for the treatment of moderate to severe depression [2], but the remission rate with the first treatment is only approximately 30% [3–5] and it is not fully understood which neurobiological mechanisms antidepressants effectively target. Escitalopram, a selective serotonin reuptake inhibitor (SSRI), has a higher affinity for serotonin transporters and less influence on other receptors than other SSRIs [6, 7]. Therefore, it can be speculated that MDD patients with a good response to escitalopram may have a pathology in which serotonergic mechanisms are highly involved. If the pathology of MDD patients with a good response to escitalopram can be clarified, it will be useful for selecting more effective treatments according to the underlying pathology.

The hippocampus plays an important role in memory formation and emotional regulation [8], and has high structural neuroplasticity, as new neurons are generated in the dentate gyrus even in adulthood [9]. Stress and depression are known to reduce the number of hippocampal neurons [10] and decrease hippocampal volume [11] through neurotoxic processes, including dysregulation of the hypothalamic-pituitary-adrenal axis [12], inflammation [13, 14], oxidative stress [15], and neurotransmitter dysregulation [11]. Changes in the function and structure of the hippocampus are considered to be significantly involved in the pathology of MDD [16–19].

Escitalopram is one of the most commonly prescribed SSRIs [20] as a first-line treatment [21, 22] for depression due to its efficacy and tolerability [23]. SSRIs are thought to promote neurogenesis and alter hippocampal volume through neurotrophic factors such as brain-derived neurotrophic factor (BDNF) [24]; however, this has not been demonstrated clearly in humans [25–27], and the involvement of other mechanisms, such as increased synaptic density [17] and anti-inflammatory effects [28, 29], has also been considered. While increases in hippocampal volume following electroconvulsive therapy have been observed consistently in many studies [30–33], these volumetric changes do not demonstrate significant associations with a clinical improvement of depressive symptoms [31, 34], but rather appear to be related to cognitive side effects [35, 36]. In contrast, there are a few reports [37, 38] of significant increases in hippocampal volume associated with SSRIs, leaving the question unresolved as to whether the antidepressant effects of escitalopram are mediated by changes in hippocampal volume.

The hippocampus exhibits structural and functional laterality [39]. Functionally, the left hippocampus is primarily responsible for context-dependent episodic memory and autobiographical memory, while the right hippocampus is more involved in visuospatial memory [40–42]. In healthy individuals, the right hippocampus tends to have a larger volume than the left hippocampus [43–45]. However, this right > left difference in hippocampal volume has been shown to change in patients with neurodegenerative conditions such as Alzheimer’s disease [46–48], epilepsy [49], and schizophrenia [50–53], suggesting a potential association with these pathophysiologies. Therefore, when investigating the effects of escitalopram on the pathophysiology of MDD, it is crucial to consider not only changes in volume but also alterations in laterality.

In this study, we hypothesized that hippocampal volume would increase more in responders to escitalopram than in nonresponders during treatment, and we investigated the relationship between escitalopram treatment response and changes in hippocampal volume and laterality.

## Methods

### Ethical approval and consent

The study was conducted in compliance with the relevant guidelines and regulations, as well as the 2008 and 2013 versions of the Declaration of Helsinki. The current study protocol was approved by the Ethics Committee of Hiroshima University. Before the administration of any experimental procedure, written informed consent was obtained from all participants.

Participants In this study, 121 patients with MDD, who were either newly diagnosed or had been continuing their visits after remission, were recruited from Hiroshima University and local clinics from March 2012 to July 2018. These patients were identified as having developed a new episode of depression and were initiated on escitalopram, in accordance with the following inclusion criteria: (a) age between 25 and 75 years; (b) outpatient status; (c) moderate to severe depressive symptoms, indicated by a score of ≥14 on the 17-item Hamilton Rating Scale for Depression (HRSD-17) [54]; and (d) diagnosis of non-psychotic MDD and current depressive episode, as determined by an experienced psychiatrist according to the Diagnostic and Statistical Manual of Mental Disorders, Fifth edition and verified through the Mini-International Neuropsychiatric Interview [55, 56] conducted by trained evaluators. The exclusion criteria were as follows: (a) diagnosis of neurological disease, current or previous psychotic disorder, current high risk of suicide, current or previous substance abuse, and serious somatic disease as determined by the Mini-International Neuropsychiatric Interview; (b) left-handedness, which was defined as a score of <0 on the Edinburgh Handedness Inventory [57]; and (c) current pregnancy or nursing. Concerns exist regarding the increased risk of suicide associated with antidepressant use in patients with MDD aged under 25 years [58]. The age cutoff of ≥ 25 years adopted in our study was not based on a formal guideline that contraindicates the use of antidepressants in younger patients. Rather, it was informed by the U.S. Food and Drug Administration boxed warning [59], which states that patients aged under 25 years are at an increased risk of suicidal ideation and behavior when treated with antidepressants. Although this warning does not constitute a formal prohibition, it has strongly influenced prescribing behavior in routine clinical practice. Consequently, to ensure patient safety and to reflect prevailing clinical practice, we set the inclusion criterion at age ≥ 25 years. Nursing was also set as an exclusion criterion. Although infant exposure to escitalopram via breast milk is considered to be clinically low [60], to avoid potential risks, many clinical trials targeting escitalopram [61–65] commonly exclude pregnant or breastfeeding women, and we adopted the same approach. Handedness may influence hippocampal volume [66]; therefore, left-handed individuals were excluded. The patients underwent magnetic resonance imaging (MRI) and HRSD-17 assessment at two time points: T1, on average 7.58 days (range: 1–14) after the initiation of escitalopram treatment; and T2, on average 54.83 days (range: 38–63) after treatment initiation. Patients who did not provide consent for the second follow-up MRI scan only received the HRSD-17 assessment at T2. For the HRSD-17, the Japanese version 1.1 [67] from the Structured Interview Guide for Combined Rating of HRSD and Inventory of Depressive Symptomatology Clinician Rated published by the Japanese Society for Psychiatric Rating Scales was used. During the T1 and T2 visits, the use of escitalopram was confirmed through prescription history and self-report. Among the patients who continued their visits after achieving remission, some were still using other psychotropic medications.

### MRI data acquisition

Structural images were acquired using 3 T MRI scanners from Siemens and GE Healthcare at four different sites (Hiroshima University Hospital: 56 subjects; Hiroshima City General Rehabilitation Center: 6 subjects; Kajikawa Hospital: 32 subjects; and Center of KANSEI Innovation: 56 subjects); details of the MRI acquisition sequences are summarized in Supplementary Table 1. Forty-five subjects scanned at Kajikawa Hospital and the Center of KANSEI Innovation underwent T1- and T2-weighted imaging. Baseline (T1) and follow-up (T2) MRI scans were obtained according to the study schedule described above.

### Image preprocessing and hippocampal segmentation

**Fig. 1:**
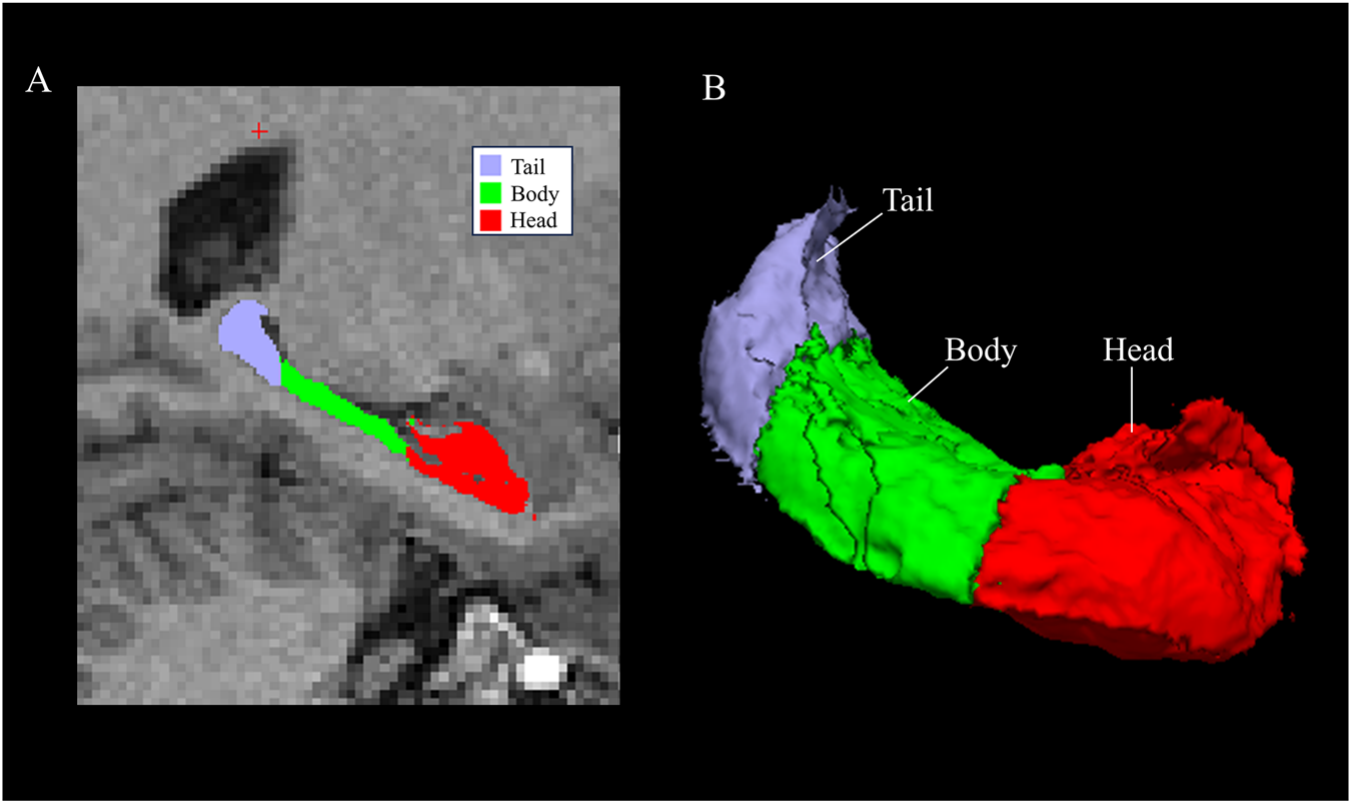
Segmentation of the hippocampus using FreeSurfer. Right hippocampal subregions presented in a sagittal magnetic resonance imaging (MRI) section (**A**). Right hippocampal subregions illustrated in a three-dimensional reconstruction (**B**).

Image processing was conducted using FreeSurfer (ver. 7.3.2) [68–70], which enables reliable measurement of total hippocampal volume and the volumes of hippocampal subregions through automated segmentation [71]. First, “recon-all” was performed on all scans. For the 45 subjects with T1- and T2-weighted images, “recon-all” was used on the T2-weighted images to improve the accuracy of segmentation. Longitudinal processing was then performed using images from one or two time points as available [72]. Longitudinal processing produced an estimate of total intracranial volume (eTIV) for each subject, which was used as a covariate for statistical analysis. Longitudinal segmentation [73] was then applied to measure the volume of the hippocampal subfields and divide the hippocampus into the head, body, and tail [68]. Figure 1 shows the automated segmentation of one patient’s hippocampus.

### Quality control

Of the 197 structural scans obtained from 121 subjects, four scans were excluded because they were defined as poor quality through manual checks and three scans were excluded due to image preprocessing failures. Hippocampal segmentation was inspected visually in each of the three orthogonal slices, and six scans were excluded because segmentation that included non-hippocampal tissue or was missing part of the hippocampus was considered a failure. The ENIGMA Group Quality Control Pipeline [74] was also referred to, and six scans with outliers outside a standard deviation of 2.698 for subfield volume or with abnormal volume ranking were excluded. After quality control screening, 178 scans from 107 subjects were analyzed further.

### Harmonization

Brain imaging datasets collected at multiple sites are subject to technical variability, known as the batch effect, which is due to systematic bias and non-biological variability caused by the use of different scanners and different imaging parameters [75]. To harmonize multicenter MRI data, the ComBat harmonization method [75, 76] was used for baseline cross-sectional data and the Longitudinal ComBat method [77] was utilized for longitudinal data at T1 and T2 (Supplementary Method). Longitudinal ComBat is a variant of ComBat that flexibly models repeated measures over time. It efficiently captures subject effects as random intercepts and incorporates time of scan into the harmonization process. By doing so, Longitudinal ComBat can detect longitudinal changes more effectively than other approaches. It has been suggested that Longitudinal ComBat should be used in studies with longitudinal data and therefore non-independent observations [78].

### Laterality index (LI)

To assess laterality in each regional volume, the LI was used, a common approach for assessing the laterality of the brain [53, 79, 80].

$${LI}=\frac{{left}-{right}}{{left}+{right}}$$

A positive LI value implies left-dominant asymmetry.

### Treatment response

Treatment response was calculated using the total score of the HRSD-17 according to the following formula:

$${Treatment\; response}( \% )=\frac{T1-T2}{T1}\times 100$$

Participants with a reduction of ≥50% in the HRSD-17 score were categorized as Responders, while those with a reduction of <50% were classified as Nonresponders.

### Six-item HRSD (HRSD-6)

The evaluation of core depressive symptoms was conducted using the HRSD-6 [81]. The HRSD-6 score was calculated by summing specific items from the HRSD-17, namely: depressed mood, feeling of guilt, work and activities, retardation, psychic anxiety, and general somatic symptoms.

### Core depressive symptoms

The HRSD-17 enables a multidimensional assessment of the diverse symptoms of depression; however, it includes items such as sexual dysfunction, sleep disturbances, and gastrointestinal symptoms, which overlap with the common side effects of SSRIs, and therefore may not accurately reflect true treatment response [82]. In contrast, the HRSD-6 focuses on core depressive symptoms and is more sensitive for detecting treatment-related changes [83]. Accordingly, Responders who achieved a reduction of at least 50% from baseline on the HRSD-6 were defined as Core Symptom Responders (CS-Responders), and those who did not meet this threshold were defined as Core Symptom Nonresponders (CS-Nonresponders). The relationship between changes in core depressive symptoms and changes in hippocampal volume was then examined.

### Statistical analysis

Statistical analyses were conducted using R (ver. 4.4.1) software, with the significance level defined as *P* < 0.05 (two-tailed). Multiple comparisons were corrected using the false discovery rate (FDR) approach with the Benjamini–Hochberg method. Demographic and clinical data were compared using chi-square tests for categorical variables and two-sample *t*-tests or Wilcoxon rank-sum tests for quantitative variables.

To compare the baseline volumes between Responders and Nonresponders, a generalized additive model (GAM) was implemented with the gamm4 package (ver. 0.2-6) [84]:

$${Volume}={\beta }_{1}\cdot s(age)+{\beta }_{2}\cdot sex+{\beta }_{3}\cdot site+{\beta }_{4}\cdot eTIV+{\beta }_{5}\cdot group$$

Age was modeled using a thin-plate spline smooth term, whereas sex, site, eTIV, and group were included as fixed effects. Age and eTIV were *z*-standardized before model fitting. In all models, the dependent variables were analyzed on their original scales to retain interpretability. Volumes are reported in cubic millimeters (mm^3^), and LI is unitless. Although we used ComBat and Longitudinal ComBat to mitigate scanner-related measurement batch effects, these procedures do not address site-level clinical heterogeneity or sampling bias. Therefore, site was included as a fixed effect to account for potential site-specific treatment effects and residual between-site variability. A similar GAM was applied to compare baseline LI between Responders and Nonresponders:

$${LI}=\,{\beta }_{1}\cdot {\text{s}}\left({age}\right)+{\beta }_{2}\cdot {sex}+{\beta }_{3}\cdot {site}+{\beta }_{4}\cdot {group}$$

To examine volumetric changes over time, we used a generalized additive mixed model (GAMM):

$${Volume}=\,{\beta }_{1}\cdot {\text{s}}\left({age}\right)+{\beta }_{2}\cdot {sex}+{\beta }_{3}\cdot {site}+{\beta }_{4}\cdot {eTIV}+{\beta }_{5}\cdot {group}+{\beta }_{6}\cdot {time\; point}+{\beta }_{7}\cdot {group}\times {time\; point}+\left(1,|,{subject\; ID}\right)$$

Subject ID was included as a random intercept. Age was modeled using a thin-plate spline, whereas sex, site, and eTIV were fixed effects. The model also included linear terms for group and time, as well as their interaction.

Changes in LI were examined with the same GAMM structure:

$${LI}={\beta }_{1}\cdot {\text{s}}\left({age}\right)+{\beta }_{2}\cdot {sex}+{\beta }_{3}\cdot {site}+{\beta }_{4}\cdot {group}+{\beta }_{5}\cdot timepoint+{\beta }_{6}\cdot {group}\times {time\; point}+\left(1,|,{subject\; ID}\right)$$

Effect sizes for each model were quantified using the approximate Cohen’s f², calculated as described in Supplementary Methods 2.

To evaluate the influence of dropout, analyses were conducted using multiple imputation and inverse probability weighting (IPW), and a complete-case analysis was performed for comparison. First, baseline predictors of dropout (age, sex, site, and group) were entered into a logistic regression model to estimate the probability of follow-up completion for each participant. These probabilities were used to construct stabilized IPW, with trimming at the 1^st^ and 99^th^ percentiles to reduce the influence of extreme weights.

Next, missing follow-up outcomes were imputed using the mice package (ver. 3.18.0) [85] (predictive mean matching, 50 imputations) incorporating baseline characteristics and outcomes. For each imputed dataset, the generalized additive model was refit with the calculated IPW as an analytic weights. Parameter estimates and standard errors across imputations were combined using Rubin’s rules. Subsequently, a complete-case analysis was conducted using generalized additive models for subjects who had data available at T1 and T2.

To examine the association between changes in core depressive symptoms and changes in hippocampal volume, a repeated measures correlation implemented in the rmcorr package (ver. 0.7.0) [86] was used:

$${Y}_{{ij}}=\mu +{\alpha }_{i}+{\beta }_{j}+{\varepsilon }_{{ij}}$$

*Y*_ij_ represents the measurement for the *i*-th subject at the *j*-th time point, μ is the overall mean, α_i_ denotes the effect specific to the *i*-th subject (individual differences), β_j_ indicates the effect at the *j*-th measurement (changes due to time or conditions), and ε_ij_ represents the error term.

## Results

### Participant characteristics and clinical outcomes

The demographic information of the participants is shown in Table 1. Fifty-three subjects (49.5%) showed a clinical response to escitalopram (≥50% reduction in the HRSD-17 score) and were classified as Responders; the remaining 54 subjects (50.5%) were classified as Nonresponders. Fifty-six subjects (52.3%) were first episode depression. Thirty-six subjects (33.6%) achieved remission (HRSD-17 score ≤ 8 at T2). There were no significant differences between Responders and Nonresponders in age, sex, BMI, eTIV, smoking, drinking, Edinburgh Handedness Inventory, verbal intelligence quotient, age of onset, depressive episodes, presence of melancholia, escitalopram dose, follow-up interval, and concomitant medications.

**Table 1:** Demographic and clinical characteristics of the patients.

	T1 (n = 107)			T2 (n = 71)		
Characteristic	Responders (n = 53)	Nonresponders (n = 54)	Statistical analysis	Responders (n = 35)	Nonresponders (n = 36)	Statistical analysis
Ethnicity (Japanese / other)	53 / 0	54 / 0		35 / 0	36 / 0	
Age, median [IQR], years	37.0 [32.0, 44.0]	42.5 [34.3, 49.8]	W = 1733, P = 0.060	37.0 [31.5, 42.0]	41.5 [33.0, 47.0]	W = 768, *P* = 0.11
Sex (male / female)	29 / 24	22 / 32	χ^2^_(1)_ = 2.09, P = 0.18	19 / 16	15 / 21	χ^2^_(1)_ = 1.13, *P* = 0.29
BMI, median [IQR], kg/m2	21.0 [19.2, 23.6]	21.8 [20.0, 24.0]	W = 1609.5, P = 0.25	21.22 [19.75, 23.98]	22.59 [20.32, 24.12]	W = 699, *P* = 0.43
eTIV, mean (SD), mm3	1510727.5 (128577.2)	1501186.5 (183160.5)	t_(95.1)_ = −0.31, P = 0.76	1539975.9 (131594.1)	1489313.0 (189264.5)	t_(62.5)_ = −1.31, *P* = 0.19
Smoking (yes / no)	20 / 33	17 / 37	χ^2^_(1)_ = 0.46, P = 0.55	15 / 20	11 / 25	χ^2^_(1)_ = 1.15, *P* = 0.28
Alcohol consumption (yes / no)	21 / 32	21 / 33	χ^2^_(1)_ = 0.015, P = 0.90	13 / 22	15 / 21	χ^2^_(1)_ = 0.15, *P* = 0.70
Edinburgh Handedness Inventory, median [IQR], L.Q.	90.0 [80.0, 100.0]	100.0 [80.0, 100.0]	W = 1454.5, P = 0.87	100.0 [80.0, 100.0]	100.0 [79.4, 100.0]	W = 651.5, *P* = 0.78
Verbal IQ, median [IQR]	111.4 [101.3, 116.4]	112.5 [105.1, 117.9]	W = 1628, P = 0.22	111.4 [101.3, 116.5]	113.9 [106.2, 120.2]	W = 770, *P* = 0.11
Age at illness onset, median [IQR], years	33.5 [28.0, 41.3]	38.0 [28.0, 45.8]	W = 1506.5, P = 0.52	33.5 [28.0, 41.0]	32.5 [26.5, 42.3]	W = 602, *P* = 0.91
Depressive episode (first / recurrent)	31 / 22	25 / 29	χ^2^_(1)_ = 0.0093, P = 0.25	20 / 15	12 / 24	χ^2^_(1)_ = 3.16, *P* = 0.076
Melancholia (yes / no)	38 / 15	45 / 9	χ^2^_(1)_ = 1.47, P = 0.23	25 / 10	30 / 6	χ^2^_(1)_ = 0.84, *P* = 0.36
HRSD17 score, median [IQR]	19.0 [16.0, 22.0]	19.5 [18.0, 23.0]	W = 1590.5, P = 0.32	5.0 [3.5, 7.0]	14.5 [11.8, 18.0]	W = 1221.5, *P* < 0.001
Escitalopram dose, mean (SD), mg	8.8 (4.2)	8.4 (4.1)	t_(104.7)_ = −0.55, P = 0.58	10.0 (5.8)	11.7 (6.8)	t_(74.6)_ = 1.2, *P* = 0.22
Follow-up intervals, mean (SD), days				46.8 (8.2)	46.3 (10.4)	t_(65.9)_ = −0.24, *P* = 0.82
Concurrent psychotropics						
benzodiazepines (yes / no)	27 / 26	37 / 17	χ^2^_(1)_ = 3.44, P = 0.07	18 / 17	26 / 10	χ^2^_(1)_ = 3.26, *P* = 0.07
antipsychotics (yes / no)	3 / 50	6 / 48	χ^2^_(1)_ = 1.032, P = 0.31	1 / 34	1 / 35	χ^2^_(1)_ = 0.00, *P* = 0.98
other SSRIs (yes / no)	3 / 50	2 / 52	χ^2^_(1)_ = 0.23, P = 0.63	2 / 33	2 / 34	χ^2^_(1)_ = 0.00, *P* = 0.97
SNRIs (yes / no)	1 / 52	2 / 52	χ^2^_(1)_ = 0.32, P = 0.57	0 / 35	2 / 34	χ^2^_(1)_ = 2.00, *P* = 0.16
NaSSAs (yes / no)	5 / 48	6 / 48	χ^2^_(1)_ = 0.082, P = 0.78	0 / 35	3 / 33	χ^2^_(1)_ = 3.05, *P* = 0.08

*BMI* body mass index, *eTIV* estimated total intracranial volume, *HRSD-17* 17-item Hamilton Rating Scale for Depression, *IQR* interquartile range, *LQ* laterality quotient, *NaSSA* noradrenergic and specific serotonergic antidepressant, *SD* standard deviation, *SNRI* serotonin and norepinephrine reuptake inhibitor, *SSRI* selective serotonin reuptake inhibitor, Verbal IQ, the Japanese version of the Adult Reading Test was used.

### Comparison of the hippocampus between Responders and Nonresponders at T1

**Fig. 2:**
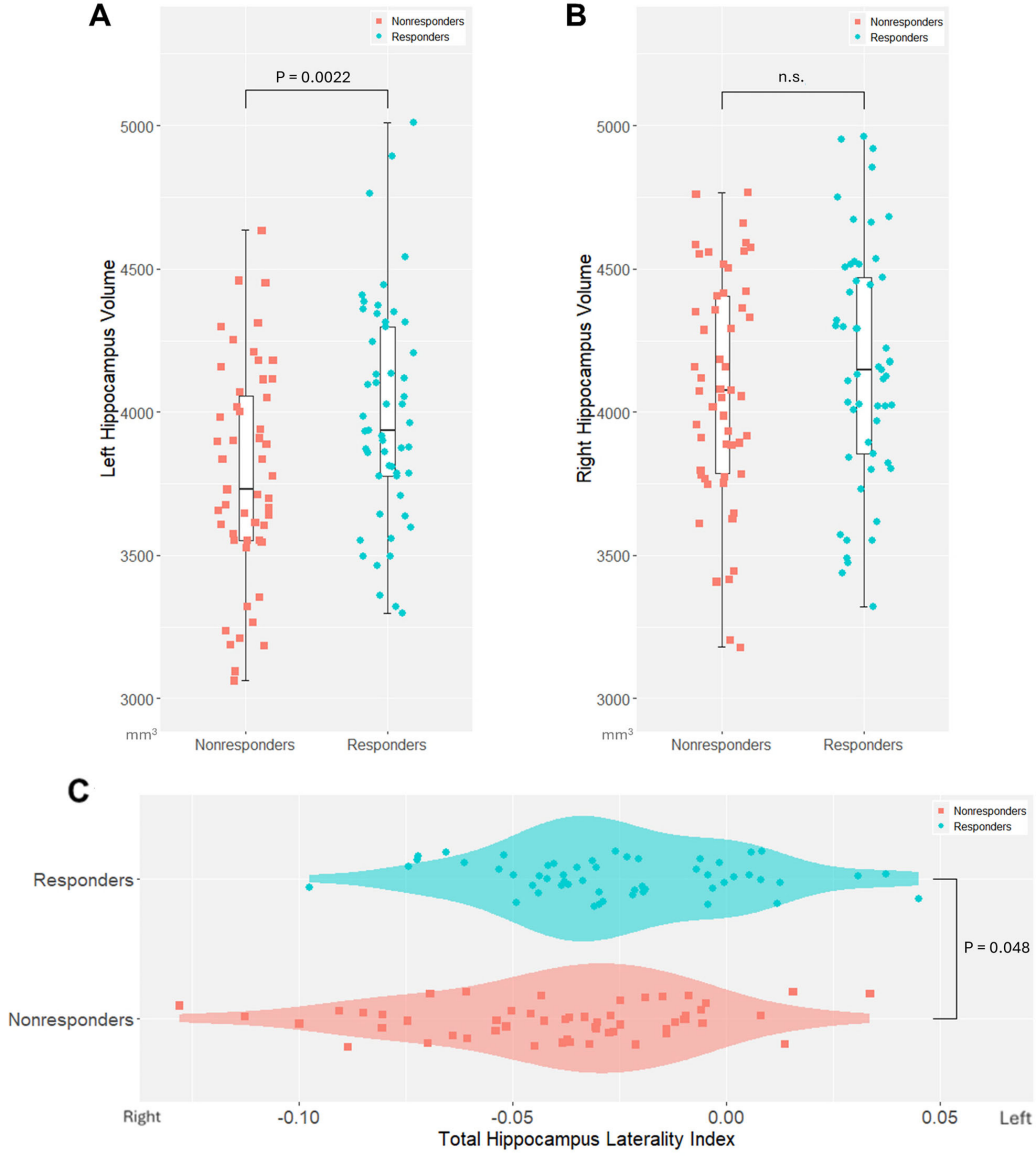
Comparison of the hippocampus between Responders and Nonresponders at T1. In the left hippocampus, Responders had greater volume than Nonresponders (**A**). In the right hippocampus, there was no significant difference in volume between Responders and Nonresponders (**B**). The laterality index was also larger in Responders than in Nonresponders (**C**). *P < 0.05. n.s., not significant, Bars reflect standard errors.

To clarify the characteristics of the hippocampus in MDD patients with a good response to escitalopram, the volume and laterality of the hippocampus were compared between Responders and Nonresponders at T1. Total volume comparison revealed that the left total hippocampus was significantly larger in Responders than in Nonresponders (β = 189.3, 95% CI [71.4, 307.2], *P* = 0.0022, Cohen’s f² ≈ 0.094; Fig. 2A). Additionally, the LI was significantly larger in Responders compared to Nonresponders (β = 0.012, 95% CI [2.2 × 10⁻⁴, 0.024], *P* = 0.048, Cohen’s f² ≈ 0.04; Fig. 2C). However, when comparisons of the LI included each subregion, no significant differences were observed after FDR correction (Supplementary Table 2).

### Comparison of structural changes in the hippocampus after escitalopram treatment

**Fig. 3:**
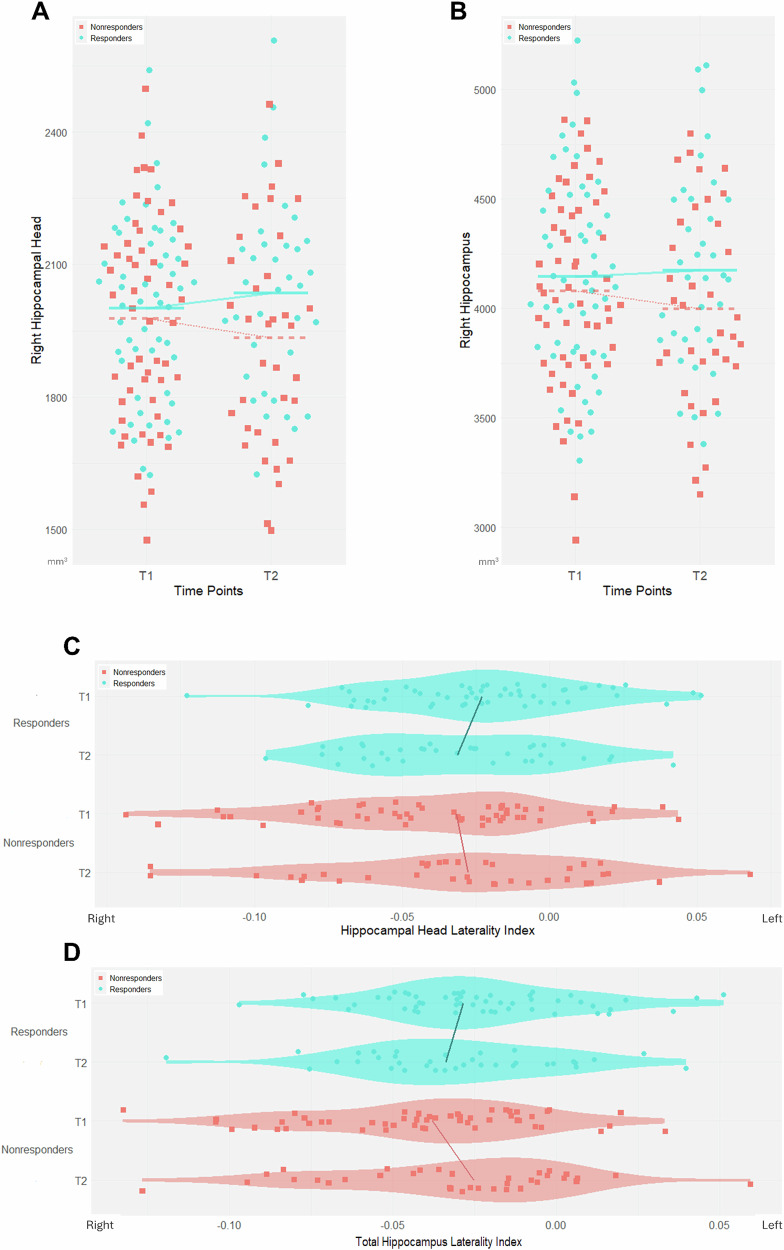
Comparison of structural changes in the hippocampus during escitalopram treatment. In the right hippocampal head (**A**) and right hippocampus (**B**), Responders showed an increase in volume after escitalopram treatment compared to Nonresponders. Additionally, in the hippocampal head (**C**), and whole hippocampus (**D**), The laterality index of Responders changed significantly rightward before and after treatment. *P (false discovery rate corrected) < 0.05.

To clarify the differences in hippocampal structural changes after escitalopram treatment, the pre- and post-treatment changes in volume and LI were compared between Responders and Nonresponders. Table 2 shows the subregions with a greater increase in volume and change in laterality in Responders than in Nonresponders. The volume of the right hippocampal head was significantly increased after FDR correction (β = 50.4, 95% CI [22.2, 78.5], FDR-corrected *P* = 0.0047, Cohen’s f² ≈ 0.006), and the volume of the right total hippocampus was significantly increased after FDR correction (β = 80.6, 95% CI [28.5, 132.8], FDR-corrected *P* = 0.011, Cohen’s f² ≈ 0.005). For the LI, the hippocampal head showed significant decreases after FDR correction (β = −3.3 × 10⁻^4^, 95% CI [−5.7 × 10⁻⁴, −8.3 × 10⁻⁵], FDR-corrected *P* = 0.038, Cohen’s f² ≈ 0.006), but the total hippocampus was no longer significant after FDR correction (Fig. 3). However, when treatment response was analyzed as a percentage, rather than when divided into Responders and Nonresponders, the LI of the total hippocampus also showed a significant decrease (β = −4.8 × 10⁻⁶, 95% CI [−8.6 × 10⁻⁶, −1.0 × 10⁻⁶], FDR-corrected *P* = 0.035, Cohen’s f² ≈ 0.009; Supplementary Table 3).

**Table 2:** Comparison of changes during escitalopram treatment in Responders and Nonresponders.

Volume	β	SE	95% CI	*P*-value	FDR *q*-value
Right hippocampal head	50.36	14.37	22.20; 78.51	0.00059***	0.0047**
Right hippocampal body	26.56	13.03	1.022; 52.10	0.043*	0.11
Right hippocampal tail	6.066	8.531	−10.65; 22.79	0.48	0.93
Right total hippocampus	80.62	26.61	28.47; 132.8	0.0028**	0.011**
Left hippocampal head	−7.62	19.97	−46.74; 31.51	0.70	0.93
Left hippocampal body	1.81	15.92	−29.40; 33.01	0.91	0.93
Left hippocampal tail	−0.87	9.39	−19.27; 17.53	0.93	0.93
Left total hippocampus	−8.61	38.03	−83.14; 65.92	0.82	0.93
**Laterality index**	**β**	**SE**	**95% CI**	***P*-value**	**FDR q-value**
Hippocampal head	−3.3 × 10⁻^4^	1.3 × 10⁻^4^	−5.7 × 10⁻^4^; −8.3 × 10⁻^5^	0.0096**	0.038*
Hippocampal body	−0.0093	0.0063	−0.022; 0.0031	0.14	0.19
Hippocampal tail	−0.0063	0.0074	−0.021; 0.0081	0.39	0.39
Total hippocampus	−0.011	0.005	−0.022; −0.00041	0.043*	0.087

*CI* confidence interval, *FDR* false discovery rate, *SE* standard error.

*P < 0.05; **P < 0.01; ***P < 0.001.

### Sensitivity analyses for attrition bias

Given the 33.6% dropout rate, sensitivity analyses were performed to assess attrition bias. No significant differences in baseline demographics or clinical characteristics were observed between completers and dropouts (Supplementary Table 4). To handle missing follow-up data, generalized additive model analyses were conducted using multiple imputation combined with IPW adjustment with baseline characteristics (Supplementary Table 5) and generalized additive model analyses were performed with completers only (Supplementary Table 6), comparing the results from each approach. While the analyses using multiple imputation combined with IPW did not reach statistical significance, the direction and magnitude of the estimates were similar, with overlapping 95% CIs.

Association between changes in core depressive symptoms and volume changes in the right total hippocampus and right hippocampal head.

To clarify whether the volume changes in the right total hippocampus and right hippocampal head were associated with changes in core depressive symptoms, repeated measures correlation analysis was conducted to examine the changes in HRSD-6 scores and volume changes pre- and post-treatment in CS-Responders and CS-Nonresponders, respectively (Table 3). The results showed that the changes in HRSD-6 scores in CS-Responders were significantly associated with changes in volume (*r* = −0.45, 95% CI [−0.70, −0.092], FDR-corrected *P* = 0.038; *r* = −0.44, 95% CI [−0.70; −0.081], FDR-corrected *P* = 0.038). Additionally, similar analysis of the changes in HRSD-17 scores and volume changes pre- and post-treatment for Responders and Nonresponders revealed a significant negative correlation in Responders (*r* = −0.40, 95% CI [−0.64, −0.079], FDR-corrected *P* = 0.027; *r* = −0.47, 95% CI [−0.69, −0.17], FDR-corrected *P* = 0.014) and a significant positive correlation in Nonresponders (*r* = 0.34, 95% CI [0.016, 0.60], FDR-corrected *P* = 0.040; *r* = 0.38, 95% CI [0.064, 0.63], FDR-corrected *P* = 0.027; Supplementary Table 6).

**Table 3:** Correlation between changes in the six-item Hamilton Rating Scale for Depression score and volume changes in the right hippocampus and right hippocampal head.

	*r*	95% CI	*P*-value	FDR *q*-value
**Right total hippocampus**				
CS-Responders	−0.45	−0.70; −0.092	0.016*	0.038*
CS-Nonresponders	0.22	−0.081; 0.48	0.15	0.15
**Right hippocampal head**				
CS-Responders	−0.44	−0.70; −0.081	0.019*	0.038*
CS-Nonresponders	0.22	−0.075; 0.49	0.14	0.15

*CI* confidence interval, *CS* core symptom, *FDR* false discovery rate.

*P < 0.05.

## Discussion

In this study, we found that Responders to escitalopram had a larger left total hippocampal volume and greater leftward laterality at baseline than Nonresponders. During treatment, the right total hippocampus and right hippocampal head volumes increased more in Responders than in Nonresponders, and the laterality of the hippocampal head shifted rightward. These volumetric changes were significantly correlated with improvements in core depressive symptoms assessed by the HRSD-6, suggesting that hippocampal structure and its plasticity are closely linked to treatment response.

Previous studies on hippocampal volume changes with SSRIs have reported mixed results [19], ranging from increases [37, 87] to no changes [88–91] or even decreases [18]. A plausible explanation for these inconsistent findings is that crucial factors such as treatment response [19], laterality, or anterior-posterior differences were often not considered. Our results highlight these specific dimensions and emphasize their importance in understanding the neuroanatomical correlates of the effects of antidepressant.

The antidepressant effects of SSRIs are thought to be mediated by various mechanisms, not only through hippocampal neurogenesis, but also through the modulation of the hypothalamic-pituitary-adrenal axis [12, 92], anti-inflammatory effects [93], and enhancement of synaptic plasticity [94–96]. Neurogenesis induced by SSRIs is considered to be gradual and of modest magnitude [97, 98], and the resulting volume changes are likely to be subtle. Importantly, not all hippocampal enlargements are beneficial, e.g., electroconvulsive therapy studies [35, 36] have shown that hippocampal volume increases can be associated with cognitive side effects, particularly memory impairments. This underscores that the functional significance of changes in hippocampal volume depends on the underlying biological mechanism and clinical context. Notably, there are reports [99] that SSRIs preferentially enhance neurogenesis in the anterior hippocampus, and reports [100] that an age-related decline in progenitor pools is more pronounced in the anterior–mid dentate gyrus compared to the posterior region, suggesting that neurogenesis is not uniform along the longitudinal axis of the hippocampus. These anterior–posterior differences indicate that the rate and regulation of neurogenesis vary across subregions, which may help explain region-specific volumetric and lateralized responses to antidepressant treatment.

The hippocampus exhibits both functional and structural laterality [39]: the left side is more involved in verbal and autobiographical memory, while the right side contributes to emotional and spatial memory [40–42]. Previous reports [89, 101, 102] suggest that larger hippocampal volumes are associated with a better treatment response and remission. In line with these observations, Responders in our study showed a larger left hippocampal volume and greater leftward laterality at baseline than Nonresponders. These structural characteristics may reflect cognitive features such as stronger verbal and autobiographical memory that facilitate a response to antidepressant treatment [103]. In contrast, the subsequent rightward shift in hippocampal head volume and laterality in Responders likely reflects a different process, i.e., improvements in stress regulation and affective disturbances mediated by right anterior hippocampal plasticity [104]. The correlation between right hippocampal changes and HRSD-6 score improvement strengthens the interpretation that right hippocampal plasticity is directly linked to the alleviation of core depressive symptoms. This dynamic pattern of baseline leftward dominance followed by treatment-related rightward enhancement suggests that the two hemispheres may play distinct roles at different stages of treatment. Molecular studies showing hemispheric differences in BDNF expression [105] and NMDA receptor distribution [106, 107] suggest that these lateralized differences have a biological basis.

Our findings also complement large-scale evidence [108] from the ENIGMA-MDD consortium, which found no case–control differences in brain structural asymmetry across more than 6700 participants. While ENIGMA indicates that asymmetry is not a general diagnostic feature of depression, our results suggest that individual differences in hippocampal laterality may instead serve as a prognostic markers of treatment response and reflect state-dependent neuroplasticity during antidepressant treatment.

This study has several strengths. It subdivided the hippocampus into six regions, enabling the simultaneous examination of laterality and anterior–posterior differences, and it focused on patients with moderate to severe depression initiating escitalopram treatment. The combined use of HRSD-17 and HRSD-6 allowed us to link hippocampal changes specifically to the alleviation of core depressive symptoms.

Nonetheless, several limitations should be acknowledged. First, because the sensitivity analysis using multiple imputation combined with IPW did not reach statistical significance, the possibility that attrition bias may have influenced the results cannot be excluded fully. However, the estimated effect was comparable in direction and magnitude to that of the main analysis. Second, the effect sizes were small. Such small values are common in longitudinal mixed-effects models due to large inter-individual variability, but the significant interaction still corresponded to divergent hippocampal trajectories between Responders and Nonresponders, supporting its clinical relevance. Finally, imaging was performed using standard-resolution MRI. It has been cautioned that automatic segmentation of the hippocampus using non-high-resolution images may compromise reliability and validity [109, 110]. To mitigate this limitation and enhance the robustness of our findings, this study categorized the hippocampus into larger regions, i.e., the head, body, and tail.

## Conclusion

This study is the first to demonstrate that increases in the volume and changes in the laterality of the right total hippocampus and right hippocampal head are involved in the treatment response to escitalopram. The response to escitalopram treatment cannot be explained fully by hippocampal volume changes alone, but it is likely that volume changes in the right hippocampus and its head play an important role in improving depressive symptoms. This study contributes to our understanding of the mechanisms of action of SSRIs and may be useful for the stratification of patients with MDD and the early detection of treatment efficacy, ultimately aiding in the development of more effective treatments in the future.

## Supplementary information

Supplementary material

## Data Availability

The MRI data from this study are registered at the SRPBS Multidisorder MRI Dataset (https://bicr-resource.atr.jp/srpbs1600/) and Brain/MINDS Beyond Human Brain MRI datasets (https://mridata-brainminds-beyond.atr.jp/), but they are not linked to data such as treatment response. For information related to treatment response, please contact the Department of Psychiatry and Neurosciences, Hiroshima University, Japan (T.K., k5105880@gmail.com or G.O., goookada@hiroshima-u.ac.jp).
