# Supplementary material for: Relationship between hippocampal volume and treatment response before and after escitalopram administration in patients with depression
Source: Transl Psychiatry. 2025 Dec 30;16:1. doi: 10.1038/s41398-025-03796-4 (PMC12783789; doi:10.1038/s41398-025-03796-4)
Supplement: Supplementary file 1 — Supplementary material [file 41398_2025_3796_MOESM1_ESM.docx]

Relationship between hippocampal volume and treatment response before and after escitalopram administration in patients with depression

**Supplementary Material**

Contents

Supplementary Method 1. Harmonization 　 2

Supplementary Method 2. Effect size quantification 4

Supplementary Table 1. Magnetic resonance imaging acquisition parameters 5

Supplementary Table 2. Comparison of the hippocampus between Responders and Nonresponders at T1 6

Supplementary Table 3. Comparison of changes in the percentage response rate during treatment 7

Supplementary Table 4. Baseline clinical characteristics between completers and dropouts 8

Supplementary Table 5. Complete-case analysis 9

Supplementary Table 6. Analysis using multiple imputation combined with inverse probability weighting 10

Supplementary Table 7. Correlation between changes in the 17-item Hamilton Rating Scale for Depression score and changes in right hippocampal volume and right hippocampal head volume 11

Supplementary References 12

**Supplementary Method 1**

In this study, the ComBat^1,2^ method was used for baseline cross-sectional data, and the Longitudinal ComBat^3^ method was used for longitudinal data before and after treatment to harmonize multicenter magnetic resonance imaging data.

ComBat (cross-sectional)

The ComBat method corrects a multivariate dataset using an empirical Bayesian estimation approach and can be used to analyze datasets obtained through different scanning procedures. This method was used to reduce potential biases and non-biological variability induced by site and scanner effects. The ComBat method utilizes the following adjustment model for location and scale:

$y_{(i,j,v)}= \alpha_{(v)}+X_{(i,j)}^{T}\beta_{(v)}+\gamma_{(i,v)}+\delta_{(i,j)}\varepsilon_{(i,j,v)}$

where $y_{(i,j,v)}$ is the hippocampal volume at scanner *i* for subject *j* and subregion *v*, $\alpha_{(v)}$ is the average volume at scanner *i* for subregion *v*, $X_{(i,j)}$ is the design matrix of subregion *v* for the biological covariates of interest (age, sex), and $\beta_{(v)}$ is the subregion-specific vector of regression coefficients corresponding to $X_{(i,j)}$. The terms $\gamma_{(i,v)}$ and $\delta_{(i,j)}$ represent the additive and multiplicative effects of scanner *i* on subregion *v*, and are unwanted terms that should be removed and follow normal $N\left( \gamma_{\left( i \right)},\tau_{(i)}^{2} \right)$ and inverse gamma $\left( \lambda_{(i)},\theta_{(i)} \right)$ distributions, respectively. $\varepsilon_{(i,j,v)}$is the error term, which follows a normal distribution with mean zero and variance $\sigma_{(v)}^{2}$. The ComBat harmonized values can be expressed as

$$y_{(i,j,v)}^{combat}=\frac{y_{\left( i,j,v \right)}-\hat{\alpha}_{\left( v \right)}-X_{\left( i,j \right)}\hat{\beta}_{\left( v \right)}-\gamma_{(i,v)}^{*}}{\delta_{(i,v)}^{*}}+\hat{\alpha}_{(v)}+X_{\left( i,j \right)}\hat{\beta}_{\left( v \right)}$$

$\gamma_{(i,v)}^{*}$ and $\delta_{(i,v)}^{*}$ are the empirical Bayes estimates of $\gamma_{(i,v)}$ and $\delta_{(i,v)}$, respectively. $\hat{\beta}_{\left( v \right)}$ and $\hat{\alpha}_{\left( v \right)}$ represent estimated coefficients associated with the biological covariates of interest and estimated population mean of subregion *v*, respectively. Model parameters are updated through empirical Bayes iterations to reduce their variance. Finally, a statistical distribution is obtained for each parameter, allowing the removal of unwanted information. Thus, ComBat simultaneously models and estimates biological and non-biological terms, and algebraically removes the estimated additive and multiplicative site effects. ComBat harmonization was performed using the R package available at

<https://github.com/Jfortin1/ComBatHarmonization.git>

Longitudinal ComBat

Longitudinal application of the Combat method, which uses random effects to account for within-subject repeated measures, has been shown to improve statistical power in longitudinal neuroimaging analysis.

The longitudinal version of the ComBat harmonization method is modeled as follows:

$$y_{\left( i,j,v \right)}\left( t \right)= \alpha_{\left( v \right)}+X_{\left( i,j \right)}^{T}{\left( t \right)\beta}_{\left( v \right)}+\eta_{(i,v)}+\gamma_{(i,v)}+\delta_{(i,j)}\varepsilon_{(i,j,v)}\left( t \right)$$

where $y_{(i,j,v)}\left( t \right)$ is the hippocampal volume at scanner *i* for subject *j* and subregion *v* at timepoint *t*, $\alpha_{(v)}$ is the average volume at scanner *i* for subregion *v* at baseline, $X_{(i,j)}\left( t \right)$ is the design matrix of subregion *v* for the potentially time-varying biological covariates (age, sex) at timepoint *t*, $\beta_{(v)}$ is the subregion-specific vector of regression coefficients corresponding to $X_{(i,j)}\left( t \right)$, $\eta_{(i,v)}$ is a subject-specific random intercept for subject *j* and subregion ν, $\gamma_{(i,v)}$ is the additive scanner *i* parameter for subregion ν, $\delta_{(i,j)}$ is the scanner *i* scaling factor for subregion ν, and $\varepsilon_{(i,j,v)}\left( t \right)$ is the error term. $\eta_{(i,v)}$ and $\varepsilon_{(i,j,v)}\left( t \right)$ follow a normal distribution with mean zero and variance $\sigma_{(v)}^{2}$. $\gamma_{(i,v)}$ and $\delta_{(i,j)}$ follow normal $N\left( \gamma_{\left( i \right)},\tau_{(i)}^{2} \right)$ and inverse gamma $\left( \lambda_{(i)},\theta_{(i)} \right)$ distributions, respectively. The Longitudinal ComBat harmonized values can be expressed as:

$$y_{(i,j,v)}^{combat}\left( t \right)=\frac{y_{\left( i,j,v \right)}\left( t \right)-\hat{\alpha}_{\left( v \right)}-X_{\left( i,j \right)}^{T}\hat{\beta}_{\left( v \right)}-\hat{\eta}_{(i,v)}}{\hat{\delta}_{(i,v)}}+\hat{\alpha}_{(v)}+X_{\left( i,j \right)}^{T}\left( t \right)\hat{\beta}_{\left( v \right)}+\hat{\eta}_{(i,v)}$$

where $\hat{\alpha}_{\left( v \right)}$, $\hat{\gamma}_{(i,v)}$, $\hat{\beta}_{\left( v \right)}$, $\hat{\eta}_{(i,v)}$, and $\hat{\delta}_{(i,v)}$ are parameter estimates.

Longitudinal ComBat harmonization was performed using the R package available at

<https://github.com/jcbeer/longCombat.git>

**Supplementary Method 2**

To quantify effect sizes, an approximate Cohen’s f² was calculated from differences in the explained variance between the “full” and “reduced” models. Analyses were performed in R with the packages mgcv^4^, lme4^5^, and MuMIn^6^.

For cross-sectional comparisons of baseline hippocampal volume between groups, ordinary linear models were used with the same covariates, modeling age with a spline basis (full model-including group; reduced model-omitting group). The coefficient of determination (*R²*) was taken from the fitted linear models, and the main-effect f² for group was computed as

$$f^{2}=\frac{R_{full}^{2}-R_{reduced}^{2}}{1{-R}_{full}^{2}}$$

For the longitudinal analyses (group × time interaction), the smooth effect of age was represented with a low-rank thin-plate spline basis (via mgcv::smoothCon, k = 3) and linear mixed-effects models (lme4::lmer) were fitted including sex, site, eTIV, time, group, group × time interaction, and a random intercept for subject. The reduced model was identical but omitted the interaction. Marginal R² (variance explained by fixed effects) was obtained following Nakagawa & Schielzeth^7^ as implemented in MuMIn::r.squaredGLMM. The interaction effect size was then

$$f^{2}=\frac{R_{full}^{2}-R_{reduced}^{2}}{1{-R}_{full}^{2}}$$

| **Site** | **Hiroshima University Hospital** | **Hiroshima City General Rehabilitation Center** | **Kajikawa Hospital** | **Center of KANSEI Innovation** |
| --- | --- | --- | --- | --- |
| *n* | 56 | 6 | 13 | 32 |
| Scanner | Signa HDxt   (GE Healthcare, Milwaukee, WI, USA) | Signa HDxt   (GE Healthcare, Milwaukee, WI, USA) | Siemens Magnetom Spectra  (Siemens, Erlangen, Germany) | Siemens Magnetom Verio (Siemens, Erlangen, Germany) |
| Magnetic field strength, Tesla | 3.0 | 3.0 | 3.0 | 3.0 |
| Number of channels per head coil | 8 | 8 | 12 | 12 |
| **T1-weighted images** |  |  |  |  |
| Sequence | SPGR | SPGR | MPRAGE | MPRAGE |
| Repetition time (TR), ms | 6788 | 6788 | 1900 | 2300 |
| Echo time (TE), ms | 1.928 | 1.928 | 2.38 | 2.98 |
| Inversion time (TI), ms | 450 | 450 | 900 | 900 |
| Flip angle, ° | 20 | 20 | 10 | 9 |
| Matrix | 256 × 256 | 256 × 256 | 320 × 320 | 240 × 256 |
| Field of view (FOV), mm | 256 × 256 | 256 × 256 | 240 × 240 | 240 × 256 |
| Voxel size, mm | 1 × 1 × 1 | 1 × 1 × 1 | 0.8 ×  0.75 ×  0.75 | 1 × 1 × 1 |
| Slice direction | sagittal plane | sagittal plane | sagittal plane | sagittal plane |
| **T2-weighted images** |  |  |  |  |
| Repetition time (TR), ms |  |  | 5000 | 6000 |
| Echo time (TE), ms |  |  | 87 | 57 |
| Matrix |  |  | 397 × 512 | 256 × 256 |
| Excitation |  |  | variable flip angle | variable flip angle |
| Voxel size, mm |  |  | 5 × 0.43 × 0.43 | 3.2 × 0.83 × 0.83 |
| Field of view (FOV), mm |  |  | 170 × 220 | 212 × 212 |
| Slice direction |  |  | transverse plane | transverse plane |

**Supplementary Table 1.** Magnetic resonance imaging acquisition parameters

**Supplementary Table 2.** Comparison of the hippocampus between Responders and Nonresponders at T1

| **Volume** | **β** | **SE** | **95% CI** | ***P*-value** | **FDR *q*-value** |
| --- | --- | --- | --- | --- | --- |
| Right hippocampal head | 31.580 | 33.87 | -34.81; 97.96 | 0.35 | 0.35 |
| Right hippocampal body | 44.10 | 26.51 | -7.867; 96.06 | 0.10 | 0.16 |
| Right hippocampal tail | 19.82 | 16.78 | -13.08; 52.71 | 0.24 | 0.27 |
| Right total hippocampus | 97.06 | 64.19 | -28.74; 222.8 | 0.13 | 0.18 |
| Left hippocampal head | 85.19 | 31.52 | 23.40; 147.0 | 0.0081** | 0.025* |
| Left hippocampal body | 64.81 | 24.41 | 16.96; 112.7 | 0.0093** | 0.025* |
| Left hippocampal tail | 34.72 | 15.94 | 3.483; 65.96 | 0.032* | 0.064 |
| Left total hippocampus | 189.3 | 60.15 | 71.40; 307.2 | 0.0022** | 0.018* |
| **Laterality Index** | **β** | **SE** | **95% CI** | ***P*-value** | **FDR *q*-value** |
| Hippocampal head | 0.014 | 0.0071 | -0.00013; 0.028 | 0.055 | 0.11 |
| Hippocampal body | 0.0091 | 0.0083 | -0.0071; 0.025 | 0.27 | 0.27 |
| Hippocampal tail | 0.015 | 0.011 | -0.0069; 0.037 | 0.18 | 0.24 |
| Total hippocampus | 0.012 | 0.0062 | 0.00022; 0.024 | 0.048* | 0.11 |

CI, confidence interval; FDR, false discovery rate; SE, standard error; **P* < 0.05; **P < 0.01.

**Supplementary Table 3.** Comparison of changes during treatment in the percentage response rate

| **Volume** | **β** | **SE** | **95% CI** | ***P*-value** | **FDR *q*-value** |
| --- | --- | --- | --- | --- | --- |
| Right hippocampal head | 0.016 | 0.0053 | 0.0056; 0.0027 | 0.0030** | 0.012* |
| Right hippocampal body | 0.0097 | 0.0047 | 0.0040; 0.019 | 0.042* | 0.11 |
| Right hippocampal tail | 0.0037 | 0.0031 | -0.0023; 0.0097 | 0.23 | 0.46 |
| Right total hippocampus | 0.029 | 0.0097 | 0.010; 0.048 | 0.0028** | 0.012* |
| Left hippocampal head | -0.0045 | 0.0072 | -0.019; 0.0096 | 0.53 | 0.73 |
| Left hippocampal body | -0.0024 | 0.0058 | -0.014; 0.0089 | 0.67 | 0.77 |
| Left hippocampal tail | -0.00065 | 0.003 | -0.0073; 0.0060 | 0.85 | 0.85 |
| Left total hippocampus | -0.0083 | 0.014 | -0.035; 0.019 | 0.54 | 0.73 |
| **Laterality index** | **β** | **SE** | **95% CI** | ***P*-value** | **FDR *q*-value** |
| Hippocampal head | -5.2 × 10⁻^5^ | 2.2 × 10⁻^5^ | -9.5 × 10⁻^6^; -9.5× 10⁻^7^ | 0.018* | 0.035* |
| Hippocampal body | -4.8 × 10⁻^6^ | 2.3 × 10⁻^6^ | -0.0000092; -0.00000040 | 0.034* | 0.045* |
| Hippocampal tail | -3.1 × 10⁻^6^ | 2.7× 10⁻^6^ | -8.3× 10⁻^6^; 2.1× 10⁻^6^ | 0.25 | 0.25 |
| Total hippocampus | -4.8× 10⁻^6^ | 1.9× 10⁻^6^ | -8.6× 10⁻^6^; -1.0× 10⁻^6^ | 0.014* | 0.035* |

CI, confidence interval; FDR, false discovery rate; SE, standard error; **P* < 0.05; ***P* < 0.01.

**Supplementary Table 4.** Baseline clinical characteristics between completers and dropouts

| **Characteristic** | **completers (n = 71)** | **dropouts (n = 36)** | **Statistical analysis** |
| --- | --- | --- | --- |
| Treatment response (Responders / Nonresponders) | 35 / 36 | 18 / 18 | χ^2^_(1)_ = 0.0, P = 1.0 |
| Age, median [IQR], years | 39.0 [33.0, 44.5] | 43.0 [35.0, 50.3] | W = 1015, P = 0.083 |
| Sex (male / female) | 34 / 37 | 17 / 19 | χ^2^_(1)_ = 0.0, P = 1.0 |
| Sites (HUH / HRC / HKH / COI) | 33 / 3 / 10 / 25 | 23 / 3 / 3 / 7 | χ^2^_(3)_ = 4.74, P = 0.19 |
| Depressive episode (first / recurrent) | 32 / 39 | 24 / 12 | χ^2^_(1)_ = 3.64, P = 0.056 |
| HRSD17 score, median [IQR] | 19.0 [16.0, 23.0] | 20.0 [18.0, 23.0] | W = 1137.5, P = 0.35 |
| Escitalopram dose, mean (SD), mg | 8.7 (4.0) | 8.6 (4.4) | t_(65.4)_ = 0.058, P = 0.95 |

COI, Center of KANSEI Innovation; HRC, Hiroshima City General Rehabilitation Center; HRSD-17, 17-item Hamilton Rating Scale for Depression; HUH, Hiroshima University Hospital; IQR, interquartile range; SD, standard deviation

**Supplementary Table 5.** Complete-case analysis

| **Volume** | **β** | **SE** | **95% CI** | ***P*-value** | **FDR *q*-value** |
| --- | --- | --- | --- | --- | --- |
| Right hippocampal head | 52.23 | 14.45 | 23.89; 80.55 | 0.00043*** | 0.0034** |
| Right hippocampal body | 28.42 | 13.09 | 2.776; 54.07 | 0.032* | 0.084 |
| Right hippocampal tail | 5.901 | 8.59 | -10.93; 22.74 | 0.49 | 0.99 |
| Right total hippocampus | 83.69 | 26.72 | 31.33; 136.1 | 0.0021** | 0.0086** |
| Left hippocampal head | -4.41 | 20.22 | -44.04; 35.21 | 0.83 | 0.99 |
| Left hippocampal body | 5.75 | 16.13 | -25.87; 37.36 | 0.72 | 0.99 |
| Left hippocampal tail | -0.14 | 9.48 | -18.71; 18.44 | 0.99 | 0.99 |
| Left total hippocampus | -0.95 | 38.48 | -76.36; 74.47 | 0.98 | 0.99 |
| **Laterality index** | **β** | **SE** | **95% CI** | ***P*-value** | **FDR *q*-value** |
| Hippocampal head | -0.014 | 0.0061 | -0.026; -0.0020 | 0.024* | 0.097 |
| Hippocampal body | -0.0089 | 0.0064 | -0.021; 0.0037 | 0.17 | 0.23 |
| Hippocampal tail | -0.0058 | 0.0075 | -0.0020; 0.0088 | 0.44 | 0.44 |
| Total hippocampus | -0.011 | 0.0056 | -0.022; 0.00015 | 0.055 | 0.11 |

CI, confidence interval; FDR, false discovery rate; SE, standard error; *P < 0.05; **P < 0.01; ***P < 0.001.

**Supplementary Table 6.** Analysis using multiple imputation combined with IPW

| **Volume** | **β** | **SE** | **95% CI** | ***P*-value** | **FDR *q*-value** |
| --- | --- | --- | --- | --- | --- |
| Right hippocampal head | 42.12 | 24.26 | -5.481; 89.71 | 0.083 | 0.28 |
| Right hippocampal body | 37.92 | 23.43 | -8.065; 83.91 | 0.11 | 0.28 |
| Right hippocampal tail | 15.141 | 20.04 | -24.25; 54.53 | 0.45 | 0.72 |
| Right total hippocampus | 97.71 | 41.18 | 16.97; 178.4 | 0.018* | 0.14 |
| Left hippocampal head | -25.11 | 30.06 | -84.11; 33.88 | 0.40 | 0.72 |
| Left hippocampal body | 5.166 | 23.37 | -40.72; 51.05 | 0.83 | 0.83 |
| Left hippocampal tail | 6.125 | 17.48 | -19.27; 17.53 | 0.73 | 0.83 |
| Left total hippocampus | -11.87 | 48.89 | -107.8; 84.02 | 0.81 | 0.83 |
| **Laterality index** | **β** | **SE** | **95% CI** | ***P*-value** | **FDR *q*-value** |
| Hippocampal head | -0.016 | 0.0072 | -0.030; -0.0020 | 0.025* | 0.055 |
| Hippocampal body | -0.013 | 0.0074 | -0.027; 0.0017 | 0.083 | 0.11 |
| Hippocampal tail | -0.0043 | 0.012 | -0.027; 0.019 | 0.71 | 0.71 |
| Total hippocampus | -0.013 | 0.0059 | -0.024; -0.0015 | 0.027* | 0.055 |

CI, confidence interval; FDR, false discovery rate; SE, standard error; *P < 0.05.

**Supplementary Table 7.** Correlation between changes in HRSD-17 and changes in right hippocampal volume and right hippocampal head volume

|  | ***r*** | **95% CI** | ***P*-value** | **FDR *q*-value** |
| --- | --- | --- | --- | --- |
| **Right total hippocampus** |  |  |  |  |
| Responders | -0.40 | -0.64; -0.079 | 0.017* | 0.027* |
| Nonresponders | 0.34 | 0.016; 0.60 | 0.040* | 0.040* |
| **Right hippocampal head** |  |  |  |  |
| Responders | -0.47 | -0.69; -0.17 | 0.0035** | 0.014* |
| Nonresponders | 0.38 | 0.064; 0.63 | 0.020* | 0.027* |

CI, confidence interval; FDR, false discovery rate; *P < 0.05; **P < 0.01.

**Supplementary References**

1 Fortin J-P, Cullen N, Sheline YI, Taylor WD, Aselcioglu I, Cook PA *et al.* Harmonization of cortical thickness measurements across scanners and sites. *Neuroimage* 2018; **167**: 104–120.

2 Fortin J-P, Parker D, Tunç B, Watanabe T, Elliott MA, Ruparel K *et al.* Harmonization of multi-site diffusion tensor imaging data. *Neuroimage* 2017; **161**: 149–170.

3 Beer JC, Tustison NJ, Cook PA, Davatzikos C, Sheline YI, Shinohara RT *et al.* Longitudinal ComBat: A method for harmonizing longitudinal multi-scanner imaging data. *Neuroimage* 2020; **220**: 117129.

4 Wood S. mgcv: Mixed GAM Computation Vehicle with Automatic Smoothness Estimation. CRAN: Contributed Packages. 2000. doi:10.32614/CRAN.package.mgcv.

5 Bates D, Maechler M, Bolker B, Walker S. lme4: Linear Mixed-Effects Models using ‘Eigen’ and S4. CRAN: Contributed Packages. 2003. doi:10.32614/CRAN.package.lme4.

6 Bartoń K. MuMIn: Multi-Model Inference. CRAN: Contributed Packages. 2010. doi:10.32614/CRAN.package.MuMIn.

7 Nakagawa S, Johnson PCD, Schielzeth H. The coefficient of determination R2 and intra-class correlation coefficient from generalized linear mixed-effects models revisited and expanded. *J R Soc Interface* 2017; **14**: 20170213.
